# Supplementary material for: Obesity Prevalence and Associated Socio-Demographic Characteristics and Health Behaviors in Russia and Norway
Source: Int J Environ Res Public Health. 2022 Aug 1;19(15):9428. doi: 10.3390/ijerph19159428 (PMC9367755; doi:10.3390/ijerph19159428)
Supplement: Supplementary file 1 [file ijerph-19-09428-s001.zip › ijerph-1820905-supplementary.pdf]

International Journal of
Environmental Research and Public Health

MDPI

SUPPLEMENTARY DATA

Table S1. Prevalence of general obesity (BMI≥30 kg/m²) by study, sex, and age.

Age, years	Men		Women	
	KYH† (*N*=1732)	Tromsø7‡ (*N*=9300)	KYH† (*N*=2389)	Tromsø7‡ (*N*=8346)
	Percent (95% CI)		Percent (95% CI)	
40-44	23.1 (18.1-29.1)	26.4 (24.2-28.7)	22.6 (18.5-27.3)	21.2 (19.3-23.2)
45-49	26.8 (21.7-32.6)	27.5 (25.3-29.7)	27.2 (22.8-32.1)	24.9 (22.9-27.0)
50-54	26.0 (21.2-31.5)	25.5 (23.4-27.9)	34.4 (29.8-39.3)	20.9 (19.1-22.9)
55-59	28.6 (23.7-34.1)	24.9 (22.7-27.3)	43.9 (39.2-48.8)	21.3 (19.4-23.5)
60-64	27.2 (22.7-32.1)	25.3 (23.0-27.7)	48.6 (43.8-53.4)	21.4 (19.3-23.6)
65-69	29.0 (24.4-34.1)	24.0 (21.7-26.6)	48.2 (43.7-52.7)	22.5 (20.2-24.9)
**Total**	27.0 (25.0-29.1)	25.7 (24.8-26.7)	38.5 (36.6-40.5)	22.0 (21.2-22.9)

BMI – body mass index. †Missing data in KYH: weight – 13 (0.3%), height – 7 (0.17%). ‡Missing data in Tromsø7: weight – 40 (0.2%), height – 39 (0.2%).

Table S2. Distribution of BMI categories according to age and study in women.

Age, years	Underweight, BMI<18.5	Normal weight, BMI 18.5-24.9	Overweight, BMI 25.0–29.9	Obesity I, BMI 30.0–34.9	Obesity II, BMI 35.0–39.9	Obesity III, BMI =>40.0	Total
			**KYH, Abs (%)**				
40-44	12 (3)	167 (48)	92 (26)	49 (14)	19 (5)	11 (3)	350 (100)
45-49	5 (1)	152 (44)	97 (28)	54 (15)	26 (7)	15 (4)	349 (100)
50-54	4 (1)	121 (32)	123 (33)	75 (20)	35 (9)	20 (5)	378 (100)
55-59	3 (1)	92 (22)	135 (33)	113 (28)	43 (10)	24 (6)	410 (100)
60-64	1 (0)	67 (16)	149 (35)	121 (29)	56 (13)	28 (7)	422 (100)
65-69	2 (0)	65 (14)	177 (38)	150 (32)	50 (11)	27 (6)	471 (100)
**Total**	**27 (1)**	**664 (28)**	**773 (32)**	**562 (24)**	**229 (10)**	**125 (5)**	**2380 (100)**
			**Tromsø7, Abs (%)**				
40-44	15 (1)	744 (44)	561 (34)	244 (15)	74 (4)	37 (2)	1675 (100)
45-49	11 (1)	726 (43)	538 (32)	277 (16)	107 (6)	38 (2)	1697 (100)
50-54	14 (1)	740 (44)	591 (35)	246 (14)	80 (5)	30 (2)	1701 (100)
55-59	10 (1)	594 (39)	606 (39)	228 (15)	78 (5)	22 (1)	1538 (100)
60-64	10 (1)	540 (38)	562 (40)	227 (16)	54 (4)	21 (1)	1414 (100)

65-69	14 (1)	449 (36)	507 (41)	217 (17)	46 (4)	18 (1)	1251 (100)
**Total**	**74 (1)**	**3793 (41)**	**3365 (36)**	**1439 (16)**	**439 (5)**	**166 (2)**	**9276 (100)**

Abs – absolute number of participants with corresponding characteristic. BMI – body mass index

Table S3. Distribution of BMI categories according to age and study in men.

Age, years	Underweight, BMI<18.5	Normal weight, BMI 18.5-24.9	Overweight, BMI 25.0–29.9	Obesity I, BMI 30.0–34.9	Obesity II, BMI 35.0–39.9	Obesity III, BMI =>40.0	Total
			**KYH, Abs (%)**				
40-44	3 (1)	66 (29)	104 (46)	39 (17)	10 (4)	3 (1)	225 (100)
45-49	2 (1)	81 (32)	103 (41)	51 (20)	14 (6)	3 (1)	254 (100)
50-54	3 (1)	80 (29)	122 (44)	55 (20)	12 (4)	5 (2)	277 (100)
55-59	3 (1)	74 (26)	130 (45)	63 (22)	13 (4)	7 (2)	290 (100)
60-64	3 (1)	89 (26)	160 (46)	76 (22)	15 (4)	3 (1)	346 (100)
65-69	4 (1)	89 (27)	144 (43)	71 (21)	20 (6)	6 (2)	334 (100)
**Total**	**18 (1)**	**479 (28)**	**763 (44)**	**355 (21)**	**84 (5)**	**27 (2)**	**1726 (100)**
			**Tromsø7, Abs (%)**				
40-44	2 (0)	339 (23)	742 (50)	293 (20)	75 (5)	20 (1)	1471 (100)
45-49	2 (0)	354 (22)	788 (50)	331 (21)	81 (5)	21 (1)	1577 (100)
50-54	2 (0)	336 (23)	729 (51)	276 (19)	68 (5)	22 (2)	1433 (100)
55-59	3 (0)	304 (22)	710 (52)	279 (21)	49 (4)	10 (1)	1355 (100)
60-64	3 (0)	334 (25)	649 (49)	269 (20)	52 (4)	12 (1)	1319 (100)
65-69	2 (0)	272 (23)	617 (53)	236 (20)	39 (3)	7 (1)	1173 (100)
**Total**	**14 (0)**	**1939 (23)**	**4235 (51)**	**1684 (20)**	**364 (4)**	**92 (1)**	**8328 (100)**

Abs – absolute number of participants with corresponding characteristic. BMI – body mass index

Table S4. Prevalence of abdominal obesity (WHR>0.9 for men and>0.85 for women) by study, sex and age.

Age, years	**Men**		**Women**	
	**KYH†** (***N*=1732**)	**Tromsø7‡** (***N*=8346**)	**KYH†** (***N*=2389**)	**Tromsø7‡** (***N*=9300**)
	**Percent (95% CI)**		**Percent (95% CI)**	
40-44	69.3 (63.0-75.0)	53.6 (51.0-56.1)	25.9 (21.6-30.8)	11.5 (10.0-13.1)
45-49	66.1 (60.1-71.7)	65.1 (62.7-67.4)	26.9 (22.5-31.8)	14.3 (12.7-16.1)
50-54	75.3 (69.9-80.0)	70.9 (68.5-73.2)	41.3 (36.5-46.3)	14.9 (13.3-16.7)
55-59	78.3 (73.2-82.7)	78.8 (76.5-80.9)	54.0 (49.2-58.8)	21.5 (19.5-23.6)

60-64	79.5 (74.9-83.4)	82.2 (80.0-84.2)	58.6 (53.9-63.2)	23.2 (21.1-25.5)
65-69	83.3 (78.9-86.9)	87.9 (85.9-89.7)	65.5 (61.1-69.7)	28.0 (25.6-30.5)
**Total**	76.1 (74.0-78.0)	72.2 (71.2-73.2)	47.0 (45.0-49.0)	18.3 (17.5-19.1)

†Missing data in KYH: waist circumference – 5 (0.1%), hip circumference – 5 (0.1%). ‡Missing data in Tromsø7: waist circumference – 77 (0.4%), hip circumference – 72 (0.4%).

Table S5. Cross-tabulation of general obesity (BMI≥30 kg/m^2) and abdominal obesity (WHR>0.9 for men and>0.85 for women) for men and women in Tromsø7 and in KYH.

**General obesity**	**Abdominal obesity, Abs (%)**		**Total**
	**Yes**	**No**	
KYH, men			
**Yes**	458 (98.3)	8 (1.7)	466
**No**	854 (67.8)	406 (32.2)	1260
Tromsø7, men			
**Yes**	2053 (96.2)	82 (3.8)	2135
**No**	3942 (63.9)	2224 (36.1)	6166
KYH, women			
**Yes**	665 (72.6)	251 (27.4	916
**No**	453 (30.9)	1011 (69.1)	1464
Tromsø7, women			
**Yes**	812 (39.9)	1122 (60.1)	2034
**No**	877 (12.2)	6332 (87.8)	7209

Abs – absolute number of participants with corresponding characteristic

Table S6. Proportions of participants with overweight (BMI 25.0-29.9 kg/m^2) among men and women in Tromsø7 and in KYH with abdominal obesity (WHR>0.9 for men and>0.85 for women) but without general obesity (BMI≥30 kg/m^2).

	**Number of participants with AO but without GO**	**Overweight, Abs (%)**
	N	**Abs (%)**
**KYH**		
Men	854	653 (76.5)
Women	453	361 (79.7)
**Tromsø7**		
Men	3942	3200 (81.2)
Women	877	663 (75.6)

Abs – absolute number of participants with corresponding characteristic
